# A reanalysis of the Institute for Research and Evaluation report that challenges non-US, school-based comprehensive sexuality education evidence base

**DOI:** 10.1080/26410397.2023.2237791

**Published:** 2023-08-07

**Authors:** Kelly VanTreeck, Shatha Elnakib, Venkatraman Chandra-Mouli

**Affiliations:** a Consultant, Department of Sexual and Reproductive Health and Research, World Health Organization, Av. Appia 20, 1202 Geneva, Switzerland.; b Full-time faculty, Department of International Health, Johns Hopkins Bloomberg School of Public Health, Baltimore, MD, USA; c Scientist, Department of Sexual and Reproductive Health and Research, World Health Organization, Geneva, Switzerland

**Keywords:** comprehensive sexuality education, CSE, sexual health education, misinformation, disinformation, evidence base, sexual education

## Abstract

Comprehensive sexuality education (CSE) prepares young people to make informed decisions about their sexuality. A review by the Institute of Research and Evaluation that analysed 43 CSE studies in non-US settings found the majority to be ineffective and concluded that there was little evidence of the effectiveness of CSE. We reanalysed the review to investigate its validity. We found several weaknesses with the review’s methodology and analysis: (1) there was an absence of a clearly articulated search strategy and specific eligibility criteria; (2) the authors put forth criteria for programme effectiveness but included studies that did not collect the data needed to show programme effectiveness and thus several studies were determined to be ineffective by default; (3) the analytical framework minimised positive intervention effects and privileged negative intervention effects; and (4) there were errors in the data extracted, with 74% of studies containing one or more discrepancies. Overall, our reanalysis reveals that the IRE review suffers from significant methodological flaws and contains many errors which compromise its conclusions about CSE. Our reanalysis is a tool for the international community to refute CSE opposition campaigns based on poor science.

## Résumé

L’éducation complète à la sexualité (ECS) prépare les jeunes à prendre des décisions éclairées concernant leur sexualité. Un examen de l’Institut de recherche et d’évaluation (IRE) qui a analysé 43 études sur l’ECS dans des pays autres que les États-Unis d’Amérique a constaté que la majorité des interventions étaient inopérantes et a conclu que l’efficacité de l’ECS n’était guère établie. Nous avons réanalysé l’examen pour évaluer sa validité. Nous avons trouvé plusieurs faiblesses dans la méthodologie de l’examen et l’analyse: (1) une stratégie de recherche clairement articulée et des critères d’éligibilité spécifiques faisaient défaut; (2) les auteurs ont proposé des critères d’efficacité des programmes, mais ont inclus des études qui n’avaient pas recueilli les données nécessaires pour montrer l’efficacité du programme et, en conséquence, plusieurs études avaient été jugées inefficaces par défaut; (3) le cadre analytique a minimisé les effets positifs et privilégié les répercussions négatives des interventions; et (4) les données extraites comportaient des erreurs, 74% des études contenant une ou plusieurs divergences. Dans l’ensemble, notre analyse révèle que l’examen de l’IRE souffre d’importants défauts méthodologiques et contient beaucoup d’erreurs qui compromettent ses conclusions sur l’ECS. Notre nouvelle analyse est un outil qui permet à la communauté internationale de réfuter les campagnes d’opposition à l’ECS fondées sur des données scientifiques médiocres.

## Resumen

La educación integral en sexualidad (EIS) prepara a las personas jóvenes para tomar decisiones informadas sobre su sexualidad. Una revisión por el Instituto de Investigación y Evaluación analizó 43 estudios de EIS en entornos fuera de EE. UU., encontró que la mayoría no era eficaz y concluyó que existía poca evidencia de la eficacia de la EIS. Reanalizamos la revisión para investigar su validez. Encontramos varios puntos débiles de la metodología y el análisis de la revisión: (1) carecía de una estrategia de búsqueda expresada claramente y de criterios de elegibilidad específicos; (2) los autores plantearon los criterios para la eficacia del programa, pero incluyeron estudios que no recolectaron los datos necesarios para mostrar la eficacia del programa, por lo cual se determinó que varios estudios eran ineficaces por omisión; (3) el marco analítico minimizó los efectos positivos y privilegió los efectos negativos de la intervención; y (4) había errores en los datos extraídos: el 74% de los estudios contenían una o más discrepancias. Proporcionamos un reanálisis exhaustivo de la revisión de la EIS por el Instituto, la cual tenía defectos graves en su metodología y estaba llena de desinformación. Nuestro reanálisis es una herramienta que la comunidad internacional puede utilizar para refutar las campañas de oposición contra la EIS basadas en mala ciencia.

## Introduction

Comprehensive sexuality education (CSE) is defined as “a curriculum-based process of teaching and learning about the cognitive, emotional, physical, and social aspects of sexuality”.^1^ CSE prepares children and young people to make informed and responsible decisions about their sexuality and lead healthy and safe lives. There is a robust evidence base and decades of programmatic learning that support CSE and its benefits for children and young people. A strong example of this is presented in UNESCO’s *International Technical Guidance on Sexuality Education, Volume 1* (2009) where 87 studies were evaluated and nearly all were found to increase knowledge, while more than one fourth improved two or more sexual behaviours.^2^ Goldfarb and Lieberman’s systematic review found support for CSE across a range of topics and ages including outcomes such as improved social/emotional learning and prevention of child sex abuse.^3^ Despite the need for CSE and the growing body of evidence in support of its effectiveness, many young people do not receive CSE. This leaves them without proper understanding of their reproductive and sexual health and with dangerous knowledge gaps.

Why CSE is not being implemented despite the large body of evidence in support of its effectiveness is in part explained by the deep-seated opposition this issue has garnered over the years. While not new, opposition to CSE has increasingly become more organised and visible.^4^ Contrary to the prevailing evidence, opponents assert not only that CSE is ineffective, but that it can cause “long-term negative effects on health”.^5^ In 2019, the Institute for Research and Evaluation (IRE), a group that identifies as “a non-profit research agency”, published a report titled “Re-Examining the Evidence for Comprehensive Sex Education in Schools: A Global Research Review” which reviewed the existing evidence on CSE and claimed to have found little evidence for the effectiveness of CSE.^6,7^ This report was published on the IRE website and in Issues in Law & Medicine; the former served as the source for our reanalysis.^7,8^ The IRE report reanalysed 43 international studies included in three authoritative reviews on CSE, including the UNESCO technical guidance, and concluded that the current evidence base does not support the effectiveness or acceptability of CSE.^2,7^ The authors reportedly found high rates of programme failure and harmful effects, and few cases of success.

Findings from the report are being used to advocate among UN member states against school-based CSE and have generated confusion and doubts about the state of the evidence on CSE. Recently, the report was used as grounds to oppose the renewal of the Eastern and Southern Africa Ministerial Commitment.^9,10^ The IRE report conclusions have been used as the basis for advocacy efforts against CSE programmes through many outlets, including expert testimonies to US legislative bodies.^6,11^

This paper reanalyses the evidence included in the IRE report to investigate the validity of its conclusions and the extent to which they reflect the body of evidence on CSE. Our manuscript is not a systematic review of CSE programmes in international settings nor is it an evidence synthesis of CSE programme impacts. Instead, it is a reanalysis and assessment of the IRE report that seeks to investigate the rigour of the methods employed in that report and the accuracy of the conclusions reached, in an attempt to explain why the report’s findings and conclusions depart from those of well established reviews that have undergone peer-review.

We have also published a commentary in the Journal of Adolescent Health (JAH) that summarises overall findings presented in this paper and discusses the implications for CSE.^12^ Unlike our JAH commentary, this manuscript provides a detailed critique of the IRE report with an in-depth explanation of our reanalysis methods and findings. Together, this manuscript and the JAH commentary complement one another in the reanalysis of the IRE report.

Our reanalysis had four objectives relating to the following four domains:

1. **Analytical framework**: To evaluate whether the analytical framework was appropriate and consistently applied.
2. **Study selection and inclusion criteria**: To determine whether the study selection process and inclusion criteria were based on standards typical of reviews.
3. **Accuracy of analysis**: To assess if the analysis was done correctly and reflected the analytical framework used.
4. **IRE Report conclusions**: To determine if the report’s conclusions flowed logically from the results.

## Methods

We retrieved 42 (of a total of 43) non-US CSE studies evaluated in the IRE international report. The US and non-US studies were separate analyses performed by the IRE. We focused on the non-US CSE studies, given that the context of CSE in lower- and middle-income countries is quite different than in the US. For our reanalysis, we used an adapted version of the AMSTAR* criteria as an organising framework.^13^ We, the authors of this manuscript, would like to acknowledge our affiliations with CSE; we work at institutions that support CSE and are advocates of evidence-based research.

To address the four objectives listed previously, the following steps were taken:

### Objective 1 (Analytical framework)

Reviews rely on collecting outcome data from the included studies to assess the effect of interventions. An analytical framework helps define the nature of the data that will be collected and provides definitions of measures of intervention effect. The IRE report used an analytical framework that helped them define programme success. We extracted their analytical framework; the operational definitions of the indicators included in the framework; their methods for collecting, coding, and reporting the data; and the process they used to determine overall programme effect. We assessed whether the analytical framework was appropriate and coherent and used it to inform our study reanalysis.

### Objective 2 (Study selection and inclusion criteria)

We attempted to reproduce the IRE search strategy by referring to the three existing research reviews the authors cited in the report as forming the basis of their study selection: (1) UNESCO’s *International Technical Guidance on Sexuality Education, Volume 1;*^2^ (2) the CDC *Community Preventive Services Task Force’s* “The Effectiveness of Group-Based Comprehensive Risk Reduction and Abstinence Education Interventions to Prevent or Reduce the Risk of Adolescent Pregnancy, HIV, and STIs”;^14^ and (3) US Department of Health and Human Services “Teen Pregnancy Prevention (TPP) Evidence Review” database.^15^ If we did not successfully find a publication in one of the three reviews, we used other academic search engines.

We then extracted the study inclusion criteria and compared them against the PICOT (population, intervention, comparison, outcome, and time) elements of the research question. These are standard components of a research question that inform pre-specified eligibility criteria upon which studies are included or excluded.^16,17^

To assess whether the review used a comprehensive and reproducible search strategy that reduces risk of selection bias, we extracted and replicated their search strategy. We also explored whether the authors conducted a critical assessment of the included studies (such as quality or risk of bias assessments^18,19^) and whether these informed their analysis and presentation of results.

### Objective 3 (Accuracy of analysis)

Data were extracted from each study and classified in accordance with the IRE analytical framework. To ensure accuracy, data extraction were conducted independently by the first and second author and their results were compared and compiled. Any discrepancies that arose in the data extraction were resolved by discussion.

We organised the results of the reanalysis into a data table similar to the one included in the IRE report to aid in comparison. We compared the data and recorded discrepancies between the IRE report and our study reanalysis. Then we performed a discrepancy analysis to quantify the scale of discrepancies. We calculated two measures: (1) percentage of studies that contained one or more discrepancies; and (2) percentage of indicators that were coded incorrectly. We also evaluated each study for overall programme effect based on the IRE analytical framework.

### Objective 4 (IRE report conclusions)

We compared the conclusions in the IRE report against the data presented in their report to determine if their conclusions flowed logically from their results.

## Results

### Analytical framework

The IRE report relied on an analytical framework that defined intervention effect in terms of 10 indicators: (1) four “key protective” indicators, (2) four “less protective” indicators and (3) two “other” indicators. Table 1 presents these indicators (column 1) and the IRE report definitions of these indicators (column 2). The classification of these indicators was used to determine each study’s overall effect.

**Table 1.** Indicator definitions and measurements used.

1	2	3
Indicator	**IRE report definition**^7^	Measurements used for undefined indicators in our reanalysis
**Key protective indicators**		
Sexual initiation	Not defined	Self-reported to be sexually active
Consistent condom use (CCU)	“Using a condom with every act of sexual intercourse”	N/A
Pregnancy	Not defined	Self-reported to be or have been pregnant
Sexually transmitted diseases (STDs)	Not defined	Biological test or self-reported history of STDs
**Less protective indicators**		
Any condom use	Not defined	Any self-reported condom use data that was not explicitly labelled as CCU
Frequent/recent sex	Not defined	Multiple measurements taken including self-reported data on: regular partner, recent sexual activity, or frequency of recent sex.
Unprotected sex	Not defined	Self-reported data on unprotected intercourse or sex without contraception (not including condom use)
# of sex partners	Not defined	Self-reported data on number of partners, having multiple partners, or mean number of sexual partners.
**Other indicators**		
Negative effects	Not defined	Any negative effect
Dual benefit	“Increasing both abstinence and condom use (by sexually active teens”	N/A

The designation of indicators as key protective indicators vs less protective indicators was not justified in the report nor was it based on evidence from prevention research that privileges some indicators over others. The report additionally lacked definitions for the included indicators: out of ten indicators, we were able to locate definitions for only two within the report. The remaining indicators were not explicitly defined, which made it difficult to reanalyse the IRE report. For example, the “unprotected sex” indicator can be defined as protection from pregnancy, from sexually transmitted infections (including HIV), or both. There is clear overlap between these interpretations and the “any condom use” indicator. UNAIDS now uses the term “condomless sex” in place of “unprotected sex” to avoid confusion with the protection from pregnancy that is provided by other means of contraception.^20^ Although we would define unprotected sex as sexual intercourse without any form of contraception, to align closely to the IRE report’s methodology we did not include condom use data for this indicator because: (1) the IRE included two other indicators that capture condom use data and (2) IRE condom use findings (continued or any condom use) did not align with their unprotected sex findings, which we took as a sign that they excluded condom use from this indicator’s definition. Column 3 in Table 1 summarises the study measurements we used for the undefined indicators in our study reanalysis. The absence of clear definitions and the ambiguity of some indicators made it difficult to interpret the results of the IRE report and to establish what data were collected for each indicator.

The authors used the 10 indicators to establish whether a CSE programme was deemed effective or ineffective. To be classified as producing a positive effect, the following criteria had to be met:^7^

- Produce a positive effect on **entire** study population (not just a subgroup); AND
- Produce a positive effect at or beyond 12 months post-programme; AND
- Produce a positive effect on one of the four key protective indicators

A CSE programme that produced a positive effect for one subgroup and not the entire population did not meet the criteria. Similarly, a programme that reduced “frequent/recent sex” and increased “any condom use” was still not classified as producing a positive effect because these were classified as less protective indicators.

In contrast, to be classified as producing a negative effect, the following criteria had to be met:^7^

- Negative effect on **any** substantial subgroup of study population; OR
- Negative effect for any duration; OR
- Negative effect on any indicator

Programmes with both positive indicator effects and negative indicator effects were classified as producing an overall negative programme effect. In addition, indicators that were outside those specified in the framework were only considered if they produced negative effects, whereas non-framework indicators for which programmes produced positive effects were not considered. For example, if a programme increased coerced sex, paid sex, or forced sex, the overall programme was classified as producing a negative effect.^21,22^ While this may be considered a reasonable classification, the same treatment was not afforded to programmes that showed positive effects for indicators that fell outside of the framework. For example, Díaz et al reported a significant increase in current use of modern contraceptive methods; however, the indicator was considered outside of the framework and so the whole study was not classified as positive.^23^ This inconsistency in approach favoured negative effects and suppressed positive programme effects.

The IRE report included a third classification of studies which they called studies showing “evidence of programme potential”. These were programmes that failed to meet the three positive effect criteria mentioned above but that (1) produced a positive effect among a subgroup; or (2) produced a positive effect for less than 12 months; or (3) produced a positive effect for one of the less protective indicators. However, while the authors did acknowledge that such programmes showcased potential, they still classified them as “failures” in their conclusions about overall CSE programme effect. Further, six indicator measurements which were classified as evidence of programme potential actually demonstrated a positive effect for the entire study population, for more than 12 months post-programme.^22,24–26^ However, because these positive effects were for one of the less protective indicators, they were not classified as positive.

### Study selection and eligibility criteria

Instead of articulating a clear search strategy, which would follow good practice, the IRE report authors stated that they relied on three existing reviews to inform their study selection. The CDC review included only US-based studies, which were outside the scope of the IRE analysis of non-US studies. UNESCO’s technical guidance included a total of 45 articles on CSE in non-US school-based settings.^2^ While there was no clear indication as to which review document each study report was extracted from, we found that 19 of the 45 eligible articles in the UNESCO review were included in the IRE report. The US Department of Health’s Teen Pregnancy Prevention (TPP) database included 14 articles on CSE in non-US school-based settings;^15^ only three of the 14 were included in the IRE’s report. The rest of the studies were not included in the IRE report. Two of the three articles were found in both the UNESCO technical guidance and the TPP database. There was thus a total of 23 studies from the IRE report that were not found in either review. Appendix 1 details which studies were found in the UNESCO review and TPP database. Of the 43 studies included in the report, we were only able to retrieve 42 study reports due to lack of citations.

It was unclear from the IRE Report how study selection took place, and why some articles were included while others were not. The authors did not document the search process in sufficient detail, which hampered our attempt to reproduce their search. With regard to the rationale for including in the IRE report the studies not included in the two reviews, the authors did not describe (1) their search strategy; (2) whether they followed established protocols for selecting studies (such as screening titles and/or abstracts and conducting full-text screenings of potentially eligible study reports); nor (3) who determined study eligibility and how discrepancies were resolved if more than one person was involved in the process. Given the polarised environment of CSE research, we did not reach out to the authors for additional information on their search strategy.

We also found that there was an absence of clearly articulated eligibility criteria which should have reflected the review’s overarching research question. The authors defined a successful programme as one producing “effects sustained at least 12 months after the programme on a key protective indicator”.^7^ The time element of their PICOT research question is thus defined by the 12 months metric and should accordingly form the basis of their inclusion criteria. Yet, many studies that were included did not take measurements at or beyond 12 months. We found that only 21 of the included study reports (50%) took measurements at or beyond 12 months. Of the remaining studies, 16 did not collect measurements at or beyond 12 months and five did not specify a follow-up period. Accordingly, 50% of the studies were ineligible, rendering them ineffective by default according to the authors’ definition of programme effectiveness. Although these studies should have been automatically disqualified from inclusion by the IRE’s analytical framework, we still evaluated these “disqualified” studies in our reanalysis to provide a thorough analysis across all objectives. Moreover, the outcomes that the authors pre-specified – one of four key protective indicators that are listed in Table 1 – were missing from two of the included studies. Those two were again rendered ineffective by default.

Additionally, the authors included all eligible studies without evidence of carrying out any quality assessments or appraisals. There are multiple ways to conduct eligibility and quality assessments; different authors of CSE systematic reviews have made decisions to include or exclude studies of low quality.^27,28^ However, the IRE authors did not describe any sort of assessment. The inclusion of all eligible studies without consideration for quality or risk of bias may very well have introduced systematic bias. We found serious heterogeneity and variation in the quality of the included studies and several of the studies had serious flaws. For example, some low-quality studies had smaller sample sizes or were purely descriptive without employing robust statistical tests. These low-quality studies were given equal weight in the IRE review to the higher quality studies. There was no effort made to either exclude them from the analysis or present a stratified analysis that accounted for study quality variation. Neither was there a narrative discussion of risk of bias anywhere in the IRE report.

### Accuracy of analysis

In the previous section, we pointed to limitations of the analytical framework put forth by the IRE. After applying the IRE framework to the body of articles included, we further identified several errors in the way indicators and studies were classified. There were gross discrepancies between the IRE report and our reanalysis in how studies were classified overall, with the IRE report identifying one additional study with negative effects and three fewer studies with positive effects. The IRE report classified indicators as producing (1) a negative effect; (2) a positive effect; (3) evidence of programme potential; (4) both positive and negative effects; (5) not significant (NS) effects; or (6) not measured (NM).^7^ Their data tables suffer from a lack of important details around effect size and statistical significance, which are critical elements of any rigorous analysis.

Our reanalysis mirrored their approach, gathering data in relation to negative effects, the four key protective indicators, the four less protective indicators, and dual benefit. We compared our findings to those presented in the IRE data table and generated a detailed report of all discrepancies between our study reanalysis and the IRE report (Appendix 2). We found that a total of 31 of the 42 (73.8%) study reports contained one or more errors.

In both the IRE report and our study reanalysis, the overall programme effect was determined by considering all 10 indicator findings and by applying the IRE analytical framework. As aforementioned, programmes with both positive indicator effects and negative indicator effects were classified by the IRE analytical framework as producing a negative overall programme effect. The IRE analytical framework only considered the overall outcome of a programme as positive or negative. To provide a more nuanced analysis, we also noted overall programme effects demonstrating evidence of overall programme potential in our study replication; the IRE did not assess this.

The overall programme effects found in our study reanalysis were compared to those found in the IRE report. This comparison is summarised in Table 2. The IRE report found that three programmes produced an overall positive effect, while we found six. Additionally, the IRE report found that eight programmes produced a negative effect, while our study reanalysis found seven. In 19 of the studies, we found evidence of programme potential (which they defined as a programme that produced positive effects either among a subgroup, for less than 12 months, or for one of the less protective indicators). These 19 programmes were entirely overlooked in the IRE analysis despite producing positive effects, as they failed to meet **all** the criteria for programme success outlined by the IRE.

**Table 2.** Overall programme effect comparison.

	1	2	3	4
	Negative effect	Positive effect	Evidence of programme potential	None
IRE	8 (19.0%)	3 (7.1%)	Not done	31 (73.8%)
Study reanalysis	7 (16.7%)	6 (14.2%)	19 (45.2%)	10 (23.8%)

### IRE report conclusions

The IRE report conclusions about international CSE programmes were compared to the IRE report data on programme effect to determine whether conclusions accurately reflected the data presented. Note, this comparison did not take into consideration the discrepancies we identified in the IRE report. Overall, we found that the report’s conclusions about the number of programmes that produced positive or negative impacts aligned with the data they presented. However, we found errors in the conclusions they made about individual indicators, including continued condom use (CCU) and any condom use. The IRE report conclusions claimed no programmes showed an increase in CCU “for any period of time or any subgroup”. However, the IRE data table presents programmes that have shown increases.^29^ Additionally, the IRE report conclusions state only one programme showed an increase in any condom use. However, the IRE report found 10 study reports that demonstrated evidence of programme potential (the best classification for a less protective indicator within the IRE framework);^25,26,30–37^ five of these measurements were taken for the entire study population.^25,30,31,33,36^

In the overall conclusions of the report, the authors state that “of the 43 studies that evaluated 39 school-based CSE programmes outside the United States, three programmes produced positive impact 12 months after the program, on a key protective outcome, for the intended population”.^7^ Later in the report, they acknowledge that of the 43 studies, only 27 actually took measurements for a key protective indicator, at least 12 months post-programme, and for the intended population, which is how they defined success in their analytical framework. According to our reanalysis, 27 is also incorrect. Only 19 study reports were on programmes that took measurements at or beyond 12 months and on key protective indicators, and three of these were for the same programme (Mathews et al), further reducing the total number of programmes satisfying these criteria to 17. These 17 studies should have been the only studies included in the analysis and should have formed the denominator for their success rates, rather than 43. According to our study reanalysis, five of these 17 programmes (29% success rate) produced a positive impact 12 months after the programme, on a key protective outcome, and for the intended population, whereas the authors concluded that they found a very high failure rate of 89%.^7^ The 89% percent rate grossly misrepresents the overall evidence, also because many of the studies were not true “failures” and actually showed evidence of programme potential according to the IRE report’s own classification. They just did not meet the stringent definition of success that the IRE used.

## Discussion

This manuscript set out to examine the validity of a recent review of CSE programmes undertaken by the Institute for Research and Evaluation.^7^ We reanalysed the report and assessed four aspects of the review’s methodology and findings: (1) analytical framework; (2) study selection and inclusion; (3) accuracy of findings; and (4) overall conclusions. Our reanalysis revealed several inconsistencies and errors in each of the four elements examined that together cast doubt on the validity of the report’s conclusions about CSE programmes in non-US settings.

With respect to the analytical framework, we found several issues with the way data were interpreted and weighed. For one, the framework lacked crucial operational definitions, which complicated our reanalysis attempts. Second, the framework privileged some indicators over others without justifying this with evidence from prevention research or behaviour change literature. This minimised positive effects and exaggerated negative effects of interventions. Moreover, no indicators that measured changes in knowledge and attitudes, which can serve as proxy for behaviour change in some cases, were included.^38–41^ In fact, 88% of studies that were included in the review measured knowledge and/or attitudes, yet none of this evidence was featured in the report.

Changes in knowledge and attitudes may or may not impact practices, depending on the broader environments in which individuals are nested – a reality that the authors of the IRE report failed to acknowledge when interpreting their results.^42^ For example, while CSE programmes may advance young people’s knowledge of safe sex, they may fail to translate into behavioural gains if young people lack access to confidential services, if contraceptive methods are not readily available, or if supportive laws and policies that enable access to contraceptives are absent. In other words, it is important to see CSE as one element in a package of interventions that includes building the knowledge and skills of young people, investing in their social networks and assets, and providing a supportive environment that includes safe and confidential health services.^43^ In reality, many adolescents and young people find that the systems and institutions around them are not geared towards meeting their needs. We would suggest that it is unrealistic to expect interventions that focus on improving adolescents’ knowledge and attitudes alone to be silver bullets if the broader environment is not conducive to behaviour change, but to exclude improvements in knowledge and attitudes as positive outcomes of an intervention is limited.

We found that the IRE report did not adhere to standards typical of scientific reviews. In terms of study selection, the report did not provide important bibliographic information for the 43 studies that were included which prevented retrieval of the full list of studies. There was an absence of a clearly articulated search strategy and lack of documentation of the exact criteria used to determine study eligibility for inclusion. Moreover, the authors included studies that did not gather data at 12 months, which was a requirement for programme success, as well as studies that did not collect data on the indicators that are needed to show programme effectiveness.

In terms of accuracy of findings, the IRE’s analysis of studies contained many errors, according to our reanalysis, as demonstrated by our finding that 74% of studies contained one or more discrepancies. Finally, the IRE report’s conclusions did not entirely align with the data they presented and inaccurately portrayed the collective body of evidence that they examined.

Taken together, our findings indicate that the IRE analysis falls short of meeting the scientific standards necessary to inform recommendations on CSE programmes.

The findings of the IRE report depart markedly from those of other reviews conducted on the topic of CSE effectiveness, which underwent a peer-review process that verified their validity and rigour. Several systematic reviews of CSE interventions have been produced over the past two and a half decades that investigate and confirm the effectiveness of CSE. For example, in 1997, Grunseit et al found that out of 47 studies that evaluated CSE programmes, 17 studies delayed sexual activity, decreased number of sex partners, or reduced unplanned pregnancy and rates of sexually transmitted diseases.^44^ In 2007, Kirby et al reviewed 83 studies which assessed the impact of sexuality and HIV education on practices among young people and found that the majority of programmes significantly improved at least one sexual behaviour; they delayed or decreased sexual behaviours or increased condom or contraceptive use.^45^ Another review conducted in 2007 by Underhill et al evaluated the impact of abstinence-plus interventions on HIV prevention and found that out of 39 trials, 23 had a positive effect on at least one sexual behaviour.^46^ In 2014, Fonner et al meta-analysed 33 studies of school-based CSE interventions to evaluate their efficacy in changing HIV-related knowledge and risk behaviours and found that students who received CSE interventions reported significantly higher condom use, fewer sexual partners, and lower rates of sexual initiation.^47^ Most recently, in 2021, Goldfarb and Lieberman’s systematic review of 80 studies that evaluated school-based CSE programmes over the past three decades found CSE programmes improved several outcomes including reductions in reports of dating violence (51 studies) and increased effectiveness of child sex abuse prevention (16 studies).^3^

### Strengths and limitations

Our reanalysis has several strengths. First, to assess the review’s methodology and reporting, we used evidence-based guidance which outlines essential practices in the conduct of scientific reviews.^48,49^ Second, two researchers independently conducted data extraction and analysis which minimised errors in reporting and reduced subjectivity. One limitation was our inability to retrieve all 43 studies included in the IRE report. This is because the authors did not provide complete and accurate bibliographic information. Nonetheless, we made serious efforts to analyse this study to the fullest extent and were able to locate 42 out of the 43 study reports. A second limitation is that we did not include independent quality assessments of the studies. This was done to maintain focus on reanalysing the IRE report which did not carry out quality assessments, but remains a significant limitation of both analyses. A third limitation is that we did not use alternative methods or frameworks to synthesise our findings. This was done, again, in an effort to stay faithful to the methods employed by the IRE in the spirit of repeating their analysis. It is critical that our study reanalysis data are not taken as an independent systematic review that reflects the CSE evidence base; our manuscript should solely be considered an analysis and critique of the IRE report. We note as a group of authors that we support the use of CSE, and the polarised nature of debate in the field meant that we were not able to contact the IRE authors for clarification where information in the report was uncertain.

## Conclusion

Our re-review was undertaken to assess the methodology and findings of the IRE Report on CSE, which concludes that there is little evidence of CSE’s effectiveness. As researchers in this field, we found this conclusion at odds with our own experience and knowledge of the evidence base. A clear body of evidence and extensive programmatic experience speak to the benefits of CSE for children and young people. Despite lack of adherence to standards of scientific rigour, the IRE’s report has been shared through news outlets and used by other anti-CSE organisations as grounds to oppose the renewal of policies on CSE programmes.^9,10,50^ The IRE is actively working with UN member states to influence policymaking and decisions around funding allocation to CSE. The IRE’s claims that the state of the evidence discredits CSE underscore the need for independent and rigorous reanalysis, a cornerstone of the scientific process. Our reanalysis sheds light on the extent of errors in the methods used by the IRE and the inaccuracies of their findings which together compromise the validity of the report’s overall conclusions against the effectiveness of CSE.

## Implications and contribution

This manuscript is the first to provide a thorough analysis of CSE misinformation research and will be a critical tool for the international community to refute CSE opposition campaigns that are becoming better organised and resourced.

## Footnotes

* A MeaSurement Tool to Assess systematic Reviews

## Appendices

### Appendix 1: Studies found in UNESCO and TPP review documents

**Table A1:** Study source list

1st author, year	Source	1st author, year	Source
Ajuwon, 2007	UNESCO	Jewkes, 2008	UNESCO
Aderibigbe, 2008	Not found	Karnell, 2006	UNESCO
Agha, 2004	UNESCO	Li, 2008	Not found
Borgia, 2005	TPP	Magnini, 2005	Not found
Cartagena, 2006	Not found	Martinez-Donate, 2004	UNESCO
Daboer, 2008	Not found	Maticka-Tyndale, 2010	UNESCO
Dente, 2005	Not found	Matthews, 2010 – Site 1	Not found
Diaz, 2005	Not found	Matthews, 2010 – Site 2	Not found
Diaz, 2005	Not found	Matthews, 2010 – Site 3	Not found
Diaz, 2005	Not found	Merakou, 2006	Not found
Diop, 2004	Not found	Okonofua, 2003	Not found
Doyle, 2010	Not found	Ross, 2007	UNESCO
Duflo, 2006	UNESCO	Smith, 2008	UNESCO
Duflo, 2015	Not found	Stanton, 1998	UNESCO
Dupas, 2011	UNESCO	Shuey, 1999	Not found
Fawole, 1999	UNESCO	Stephenson, 2008	TPP, UNESCO
Fitzerald, 1999	TPP, UNESCO	Taylor, 2014	Not found
Harvey, 2000	Not found	Thato, 2008	UNESCO
Henderson, 2007	UNESCO	Visser, 2007	Not found
James, 2006	UNESCO	Walker, 2006	UNESCO
Jemmott, 2015	Not found	Wight, 2002	UNESCO
Jewkes, 2008	UNESCO	Ye, 2009	Not found

### Appendix 2: Detailed discrepancy report

**Table B1:** Discrepancy summary

Study	Measurement	Study Replication Findings	IR&E Report Findings
Aderibigbe, 2008	Any condom use	Evidence of programme potential at 3 months. Table 5 indicates that pre-intervention data demonstrated no significant difference amongst groups (*p* = 0.8761). Study participants who used condoms increased by 36.5% and control participants who used condoms increased by 1.8%. The post-intervention data were significantly different (*p* = 0.0003).	Report indicated that this was measured at 3 months, but did not colour code it to indicate the impact.
Aderibigbe, 2008	Frequent/recent sex	Evidence of programme potential at 3 months. Table 3 reports the number of sexual partners in the past 3 months (recent sex). The number of participants who reported 0 partners increased from 8 to 30 in the study group and decreased from 11 to 8 in the control group. The pre-intervention values were not significant (no difference among groups), but the post-intervention values were significant (*p* = 0.0002). This demonstrates the significant positive effect of the intervention on recent sex. Data were measured for Frequency of Sex (Table 4), but it was not significant.	Reported as NS.
Aderibigbe, 2008	# sex partners	Evidence of programme potential at 3 months. Table 3 reports the number of sexual partners in the past 3 months. The % of study participants who reported 2-4 partners decreased by 20.3% while the % of control participants increased by 1.7% (pre intervention showed no significant difference, post intervention *p* = 0.0431). The % of study particpants who reported >4 partners increased by 0.2% and the % of control participants increased by 3.7% (pre intervention *p* = 0.0049, post intervention *p* = 0.0011).	Reported that there were data at 3 months, but did not colour code it to indicate the impact.
Agha, 2004	Frequent/recent sex	NS. Table 6, “Has regular partner” data are not significant, *p* > 0.05.	Reported as evidence of programme potential at 6 months.
Ajuwon, 2007	Any condom use	NS. Tables 3 and 4 report the condom use baseline and post-intervention measurements. Respectively, *p* = 0.96 and *p* = 0.51.	Reported as evidence of programme potential.
Ajuwon, 2007	Sexual Initiation	Positive and negative results at 9 months. Teacher instruction (E1) and mixed intervention (E3) had a lesser increase than the control while the peer led intervention (E2) did not. Proportion that had ever had sex: E1 0.4% increase, E2 9.8% increase, E3 4.6% increase; Control 5.7% increase. *P* values for baseline and post-intervention: 0.022 and 0.0034, respectively.	Reported as NM.
Borgia, 2005	Negative Effect	Negative effect. Yes, condom use and unprotected sex.	Reported as no.
Borgia, 2005	CCU	Negative effect. Decrease in always using condoms: Teacher led from 29.0 to 22.3%, Peer led from 36.8 to 27.6% (*p* < 0.05).	Reported as NM.
Borgia, 2005	Any condom use	Negative effect. Figure 1 shows the changes in condom use for both the teacher and peer interventions. While both interventions saw more participants responding to the condom use survey question with “sometimes use a condom”, this was due to a decrease in “always use a condom”. This data were significant (*p* < 0.05).	Reported as NS.
Borgia, 2005	# sex partners	Negative effect. Teacher intervention had significant increase in respondents who reported having more than one partner (Figure 1, *p* < 0.01). Peer intervention was not significant.	Reported as NS.
Dente, 2005	Frequent/recent sex	Evidence of programme potential. Study states: “students of group 1 and group 2 were less likely to have had intercourse with casual partners than those of group 3 (*p* = 0.006)”. Groups 1 and 2 are the intervention groups; group 3 is the control group.	Reported as NM.
Dente, 2005	Sexual Initiation	Positive effect. Table 2 presents data of who have ever had sex in their lifetime. Voluntary Counselling and Testing (VCT) & School Health Education (SHE): 31.4%, SHE: 57.1%, Control: 62.9%. *p* < 0.001.	Reported as NS.
Diaz, 2005; Salvador	Unprotected sex	Evidence of programme potential. Table 6 reports an AOR (study:control) of 2.82 (95% CI: [1.45–5.49]) for current use of modern contraceptive methods. Note, this was at no specified time period after the intervention because these groups were identified from actual school settings.	Reported as NM.
Doyle, 2010	Any condom use	Evidence of programme potential at 3 years in subgroup. Table 3 reports a higher condom use with non-regular partner among females in the intervention compared to those in the control (aPR 1.34 *p* < 0.05).	Reported as NS.
Doyle, 2010	# sex partners	Evidence of programme potential at 3 years in subgroup. The intervention was associated with a reduction in the proportion of males reporting more than four sexual partners in their lifetime (aPR 0.87, 95%CI 0.78–0.97)	Reported as NS.
Duflo, 2006	Pregnancy	NS. Table 6 reports childbearing rates. Data for “has started childbearing” is not significantly different between the teacher training and reducing the cost of education programmes.	Reported as NM.
Duflo, 2006	# sex partners	NM	Reported as NS.
Duflo, 2015	Sexual Initiation	NS. Table 6 reports long-run impacts on participants’ responses to “Ever had sex”. Data were only significant at 10% level.	Reported as NM.
Duflo, 2015	Any condom use	NS. Table 5 presents data for “Used condom at last sex” but *p* > 0.05.	Reported as NM.
Dupas, 2011	Pregnancy	NS. Table 3 reports the childbearing probability. The RR data are not significant at a 95% CI (*p* = 0.10). The TT data do not have any significant childbearing data.	Reported as positive and negative effect.
Dupas, 2011	Frequent/recent sex	NS. Table 6 presents information for “Currently has regular partner”. I would say that this qualifies as frequent/recent sex as this context is refering to regular sexual partner.	Reported as NM.
Fitzgerald, 1999	Any condom use	Evidence of programme potential at 6 months. Table 5 reports data for condom use (used condom). The difference in condom use between intervention and control youth at follow-up (78% vs. 64%) was very similar to that at baseline (78% vs. 67%), although the difference at follow-up reached statistical significant (*p* < .05).	Reported as negative effect.
Fitzgerald, 1999	Dual benefit (12 months)	NS. Due to the discrepancy for Fitzgerald CCU, this is also a discrepancy. Sexual initiation was measured but was NS.	Reported as NM.
Fitzgerald, 1999	CCU	NS. Among intervention youth, the percentage of youth reporting frequent use of condoms (“always”/“usually”) increased 16% (from 61% at baseline to 77% at follow-up) compared to only a 4% increase (64% to 68%) among control youth (*p* = not significant).	Reported as NM.
James, 2006	Sexual Initiation	NM. The study does not discuss data on sexual initiation. Additionally, the original report marks this as programme potential at 4 months, but there is only f/u at 6 and 10 months.	Reported as measured at 4 months; coded as evidence of programme potential.
James, 2006	Any condom use	Evidence of programme potential at 6 months. The students in the full implementation group reported significantly more condom use at last sex (B = –.80, SE = .40, Wald (1, 157) = 4.16, *p* < .05, OR = .45). The original report is misleading because the group that showed positive impact was the full implementation group. This was compared against the control group. This aligns with the original report's definition of programme potential because it was measured at 6 months, but it was still misreported that it reflected a subgroup.	Reported as evidence of programme potential: “at Prog End-Subgroup O”.
James, 2006	Frequent/recent sex	Evidence of programme potential at 6 months. The students in the full implementation group reported significantly less sexual activity in the previous 6 months (B = –.53, SE = .24, Wald (1, 657) = 4.98, *p* < .05, OR = .59). The original report is misleading because the group that showed positive impact was the full implementation group. This was compared against the control group. This aligns with the original report's definition of programme potential because it was measured at 6 months, but the original report misreported that it reflected a subgroup.	Reported as evidence of programme potential: “at Prog End-Subgroup O”.
Jemmott, 2015	Frequent/recent sex	Positive effect. Table 3 reports the odds ratios for vaginal intercourse in the past 3 months. The overall intervention effect was not significant (*p* = 0.076), but the short term intervention effect (3, 6, 12 month) had an OR = 0.62 (*p* = 0.022). The insignificant overall follow up does not discount the significant data at 12 months.	Reported as NS.
Jemmott, 2015	# sex partners	Positive effect. Table 3 reports the odds ratios for multiple partners in the past 3 months. The overall intervention effect was not significant (*p* = 0.095), but the short term intervention effect (3, 6, 12 month) had an OR = 0.50 (*p* = 0.0180) (considered 12 months post-program).	Reported as NS.
Jemmott, 2015	STDs	Positive effect. The intervention group had significantly reduced curable STIs at 42-month follow-up, OR = 0.71, 95% CI [0.54, 0.95], but not at 54-month follow-up, OR = 1.15, 95% CI [0.84, 1.57]. The insignificant 54-month follow up does not discount the significant data at 54 months. These data reflect the entire intervention group, so stating subgroup is incorrect. Because the data are reported at 42 months, this aligns with the definition of positive effect.	Reported as evidence of programme potential at 42 months, noting “Subgroup O”.
Jewkes, 2008	STDs	Positive effect. Table 3 reported significantly lower incidence of HSV-2 in Stepping Stones than control intervention for both men and women (incident rate ratio: 0.67, *p* = 0.036). There was lower incidence of HIV in Stepping stones than control, but was not significant. These data were normalised across both follow-ups at 12 and 24 months.	Reported as positive and negative effect.
Jewkes, 2008	Frequent/recent sex	NM. Recent or frequent sex was not measured in this study. There was a negative impact for transactional sex, which is captured in the “Negative effect” column. Transactional sex is not recent sex. This is misleading and incorrect.	Reported as a negative effect.
Jewkes, 2008	Dual benefit (12 months)	NM. Sexual initiation is not measured (correctly marked by report). Therefore, this should be reported as NM too.	Reported as NS.
Karnell, 2006	Sexual Initiation	NM. Table 2 measures this at the baseline. It is included in other tables to analyse data by those who had and hadn’t had sex at the pretest, but it is never re-measured after the intervention.	Reported as NS.
Li, 2008	Frequent/recent sex	NS. Table 2 reports results of whether participants have engaged in sexual intercourse in the past 6 months but *p* > 0.05.	Reported as NM.
Li, 2008	Sexual Initiation	NM	Reported as NS.
Magnani, 2005	Overall comment	Spelled the last name wrong	Spelled “Magnini”
Martinez-Donate, 2004	Any condom use	NM. Condom acquisition was measured but use was not.	Reported as NS.
Mathews, 2010	Overall comment	Spelled the last name wrong	Spelled “Matthews”
Mathews, 2010 – Site 3 (DarEsSalaam)	Sexual Initiation	Positive effect. Table 3 presents adjusted odds ratio for sexual debut during the 12–15 month study to be 0.65 (*p* > 0.05) showing that study participants in this group are significantly less likely to have initiated sexual activity. This was clearly a positive impact and was measured for the entire study population (not a subgroup).	Reported as evidence of programme potential and indicated for subgroup.
Merakou, 2006	Sexual Initiation	Evidence of programme potential at 8 months. Table 4 presents responses to statement “I never had sexual relationships”. The intervention group showed a positive effect decreasing by 14.2% (*p* = 0.001) and the control group decreasing by 10.5% (*p* = 0.064) but it was not significant. This confirms that the intervention group had a significant positive impact on sexual initiation.	Reported as negative effect.
Merakou, 2006	Any condom use	Evidence of programme potential at 8 months. Table 4 shows responses to statement “During the last year I initiated the use of a condom with my partner.” In the intervention group agreeance to this statment increased by 1.3% (*p* = 0.017) where as the control group’s agreeance decreased by 5.4% but was not significant. The measurement was taken 8 months post baseline (Oct 1997 to May 1998).	Reported as evidence of programme potential for 6 months post baseline.
Merakou, 2006	Negative Effect	No. See error for Merakou, 2006: sexual initiation.	Reported negative effect for sexual initiation.
Merakou, 2006	Dual benefit (12 months)	Evidence of programme potential at 8 months. See sexual initiation and any condom use.	Reported as NM.
Okonofua, 2003	Any condom use	Evidence of programme potential at 11 months. Table 4 reports the odds ratios for condom use. The odds ratio for increased condom use pre- to post-intervention within study groups between both sexes was OR = 1.48 (95% CI = 1.22–1.79) in the intervention group and OR = l.ll (95% CI = O.86–1.42) in the two control groups combined.	Reported as evidence of programme potential at 1 year for a subgroup.
Okonofua, 2004	STDs	Evidence of programme potential at 11 months. Table 6 reports intervention vs. both controls both genders adjusted change for STD symptoms as 0.68 (significant at 95% CI) meaning that intervention participants were 0.68 times as likely as control participants to report STD symptoms.	Reported as positive effect at 1 year.
Ross, 2007	# sex partners	Evidence of programme potential for a subgroup at 3 years. Significant effect in males but not females. Table 4 shows RR with 95% CI for reporting more than 1 partner in past 12 months. Males: 0.69 (0.49, 0.95), positive intervention effect. Females: 1.04 (0.58, 1.89), not significant. Data demonstrate positive effect and insignificant data.	Reported as positive and negative effect.
Ross, 2007	Any condom use	Evidence of programme potential for a subgroup at 3 years. Significant effect in males but not females. Table 3 shows RR with 95% CI for used condom at last sex. Males: 1.47 (1.12, 1.93), positive intervention effect. Females: 1.12 (0.85, 1.48), not significant. Data demonstrate positive effect and insignificant data.	Reported as positive and negative effect.
Shuey, 1999	Dual benefit (12 months)	NM. Condom use is NM and was reported correctly, therefore dual benefit should be reported as NM.	Reported as NS.
Stanton, 1998	Sexual Initiation	Positive effect. Table 1 shows that the number of remaining virgins (among those who were virgins at baseline) in intervention group was 17% (*p* < 0.05) and the number of remaining virgins in the control group was 9% (not significant) 12 months post-intervention. This demonstrates that the intervention had a significant positive effect on sexual initiation, whereas the control group did not. The measurement of remaining virgins was taken for the entire study population (not a subgroup).	Reported as evidence of programme potential at 12 months for a subgroup.
Stanton, 1998	Any condom use	Evidence of programme potential for a subgroup immediately post-intervention. Baseline virgins. Table 3 shows that the 6 and 12 mo f/u are not significant.	Reported as evidence of programme potential at 6 months for a subgroup.
Stephenson, 2008	STDs	NS. Table 2 and 3 measure ever been told by a doctor or nurse that you have an STD but results were NS.	Reported as NM.
Taylor, 2014	Any condom use	Evidence of programme potential at 8 months. Table 2 reports the intervention effect on condom use to be positive; 0.98 (*p* < 0.01).	Reported as evidence of programme potential at 5 months.
Taylor, 2014	Dual benefit (12 months)	NS. Condom use and sexual initiation were both NS.	Reported as NM.
Visser, 2007	Sexual Initiation	Positive effect. Table 3 reports that the intervention group sexual experience remained the same 18-months post-intervention (no significant change). The control group sexual experience increased significantly (*p* < 0.05). This demonstrates a positive intervention effect because the intervention significantly reduced sexual initiation.	Reported as positive and negative effect.
Visser, 2007	Frequent/recent sex	Evidence of programme potential at 18 months. Table 3 presents data for having had sex in the past 3 months. The intervention group did not see a significant change, but the control group saw a significant increase (*p* < 0.001) in recent sex.	Reported as positive and negative effect.
Walker, 2006	Frequent/recent sex	NS. Table 1 compares participants in each group who have been sexually active. It is not explicitly defined to be initiation, so it is assumed it is for recent sex.	Reported as NM.
Wight, 2002	Dual benefit (12 months)	NS. Any condom use and sexual initiation were both NS.	Reported as NM.
Ye, 2009	Any condom use	Evidence of programme potential at 1 month. The study measured condom use if intercourse happened. At one month follow-up there was a significant increase of condom use in the intervention group (*p* < 0.01). There was no significant effect between one month and one year follow-up.	Reported as NS.
